# Protein drift-diffusion in membranes with non-equilibrium fluctuations arising from gradients in concentration or temperature

**DOI:** 10.1371/journal.pcbi.1013678

**Published:** 2025-11-21

**Authors:** Dev Jasuja, Paul J. Atzberger

**Affiliations:** Department of Mathematics, Department of Mechanical Engineering, University of California Santa Barbara (UCSB), Santa Barbara, California, United States of America; University of California Riverside, UNITED STATES OF AMERICA

## Abstract

We investigate proteins within heterogeneous cell membranes where non-equilibrium phenomena arises from spatial variations in concentration and temperature. We develop simulation methods building on non-equilibrium statistical mechanics to obtain stochastic hybrid continuum-discrete descriptions which track individual protein dynamics, spatially varying concentration fluctuations, and thermal exchanges. We investigate biological mechanisms for protein positioning and patterning within membranes and factors in thermal gradient sensing. We also study the kinetics of Brownian motion of particles with temperature variations within energy landscapes arising from heterogeneous microstructures within membranes. The introduced approaches provide self-consistent models for studying biophysical mechanisms involving the drift-diffusion dynamics of individual proteins and energy exchanges and fluctuations between the thermal and mechanical parts of the system. The methods also can be used for studying related non-equilibrium effects in other biological systems and soft materials.

## Author summary

We introduce theoretical frameworks and computational simulation methods for modeling and investigating protein dynamics within heterogeneous membranes in non-equilibrium regimes. A hybrid discrete-continuum approach is developed allowing for tracking individual proteins and their coupling to spatial variations in concentration and temperature captured by continuum fluctuating fields. Investigations are performed of biological processes and related phenomena arising from fluctuations, gradients in concentration, variations in temperature, and other non-equilibrium effects.

## Introduction

Cellular membranes are heterogeneous mixtures of proteins, lipids, and other small molecules [1–6]. The spatial-temporal organization of membrane proteins and their kinetics play important roles in biological functions [7–10]. This often involves non-equilibrium processes [11–13] and related phenomena breaking detailed-balance [14,15]. This includes active transport by motor proteins [16–18], mitochondrial ATP synthesis [19–21], ion exchanges by pumps and facilitated diffusion [1,22], active cytoskeletal forces [23,24], and other mechanisms [1,11]. A fundamental challenge in studying many of these cellular processes and related *in vitro* experimental systems is to gain insights into the membrane proteins and their spatial-temporal distribution and kinetics. This is impacted by drift-diffusion dynamics and reactions coupled to signals originating both within the cell and from the surrounding environment [1,25–27]. Cellular and environmental factors can include local variations and gradients in concentrations of signaling molecules, such as in chemotaxis and cell-cell communication [28–30]. Additional variations also can arise from sources or sinks of heat or chemical reactions to yield gradients in external temperatures, serving as important signals in thermotaxis, reproduction, and cell homeostasis [31–34]. Recent *in vitro* experiments also have been developed to probe the interactions of proteins and synthetic particles with local concentration and temperature gradients, and to manipulate them at the single-molecule level [35–41].

Capturing non-equilibrium effects important in these biological systems and experimental assays poses several challenges for theoretical modeling and practical simulation. This includes the need to capture the drift-diffusion dynamics of proteins as they move through regions in the membrane having different concentrations, temperatures, or other types of heterogeneity. In addition to the dynamics of the protein motions, the concentration and temperature fields can change over time from exchanges of mass and energy. For continuum mechanics descriptions at such spatial-temporal scales, both local concentrations and temperatures can spontaneously fluctuate from under-resolved smaller-scale exchanges.

Most theoretical modeling and simulation methods treat homogeneous systems. This includes Brownian-Stokesian Dynamics [42,43], Coarse-grained approaches with Langevin thermostats [44–46], and continuum mechanics formulations such as Stochastic Immersed Boundary Methods (SIBMs) [47,48], related fluctuating hydrodynamic approaches [49–51], and Stochastic Eulerian Lagrangian Methods (SELMs) [52,53]. These computational simulation methods are based on continuum hydrodynamic descriptions and statistical mechanics primarily in regimes at thermodynamic equilibrium.

Related work treating non-equilibrium regimes include recent theoretical and simulation studies of Hot Brownian Motion and Soret effects that treat diffusion of particles within temperature gradients [54,55]. This has been modeled by using temperature dependent viscosities [55], renormalized diffusivities [37], or through molecular dynamics simulations of a particle in Lennard-Jones fluids [56–58]. There also has been some work using fluctuating hydrodynamics to derive generalized Langevin equations for Hot Brownian Motions for translational and rotational motions [57,59] and for proteins that can react to or generate curvature in membranes [60,61]. Most of these approaches assume regimes with time-scale separation where they can reduce the descriptions to effective tensors for a single particle and do not track environmental changes in the temperature or concentration fields and their fluctuations.

We develop here non-equilibrium statistical mechanics approaches that use hybrid discrete-continuum descriptions. We capture both the individual protein drift-diffusion dynamics and the spatial-temporal evolution and fluctuations arising from spatially varying concentration and temperature fields within the membrane. We couple proteins tracked at the single-molecule level with these fields. We also develop stochastic numerical methods to obtain practical simulation approaches. We consider in this initial work the case when the membranes are treated as static without shape undulations where the geometry remains approximately flat. We develop methods for capturing non-equilibrium effects impacting the drift-diffusion dynamics of individual proteins within heterogeneous environments.

We demonstrate how our approaches can be used to investigate phenomena in membrane-protein systems that include (i) how concentration gradients and kinetics drive the spatial organization of proteins, (ii) the roles of fluctuations in the encoding of signals by proteins to sense external thermal gradients, and (iii) how localized laser heating, as in *in vitro* experiments, can probe and impact protein escape kinetics from local energy wells within heterogeneous membranes. Our introduced simulation methods also can be used to investigate other non-equilibrium phenomena for systems where significant roles are played by particle drift-diffusion dynamics coupled to local variations and fluctuations in concentration or temperature.

## Materials and methods

### Membrane-protein system: Stochastic non-equilibrium model

We model the membrane and proteins using a hybrid stochastic continuum-discrete approach. For the drift diffusion dynamics of an individual protein, we use

$$\frac{d𝐗}{dt}={𝐌}_{XX}{𝐅}_{X}+k_{B}\theta_{P}\left(\nabla_{X}\cdot {𝐌}_{XX}\right)+{𝐇}_{thm,X}.\;\;$$ (1)

The protein location within the membrane is denoted by 𝐗, the forces by ${𝐅}_{X}$, the temperature by $\theta_{P}$, and thermal fluctuations by ${𝐇}_{thm,X}$. The over-damped kinematic hydrodynamic response to an applied force is given by the mobility ${𝐌}_{XX}$ [43,44,62]. The *k*_*B*_ denotes the Boltzmann constant [63].

For convenience, we also alternatively will refer to the combined contributions of the fluctuations to the protein using notation ${𝐆}_{thm,X}=k_{B}\theta_{P}\left(\nabla_{X}\cdot {𝐌}_{XX}\right)+{𝐇}_{thm,X}$. The divergence term in Eq 1 arises from the diffusivity depending on the mobility ${𝐌}_{XX}$ which has a spatial dependence [62,63]. In the case of conservative forces, we have ${𝐅}_{X}=-\partial_{X}U$. We discuss how the thermal fluctuations are determined and other details below.

For tracking the concentration of a chemical species within the membrane, we use the continuum field *c*(*x*, *t*) = *c*_0_*q*(*x*, *t*). The *q* has the dynamics

$$\frac{\partial q(x,t)}{\partial t}=\text{div}\left(\bar{\kappa }\nabla q(x,t)\right)-\text{div}\left(-\frac{1}{\gamma }q(x,t)\nabla \Phi (x;𝐗)\right)+{𝐠}_{thm,q}.\;$$ (2)

The total concentration is given by *c*_0_ and *q*(*x*,*t*) gives the spatial distribution of the chemical species within the membrane. This yields at time *t* and location *x* the concentration *c*(*x*, *t*) = *c*_0_*q*(*x*, *t*) for a membrane chemical species. The diffusivity is denoted by $\bar{\kappa }$ and satisfies the Stokes-Einstein relation $\bar{\kappa }=\theta_{C}/\gamma$ [63]. The $\theta_{C}$ denotes the temperature of the membrane and *γ* denotes the hydrodynamic drag of the molecular species. The fluctuations are given by ${𝐠}_{thm,q}$. The Φ denotes the chemical potential for the molecular species at location *x* given the protein is at location 𝐗. We consider the case here of a flat membrane and model the spatial fields of the system as having periodic boundary conditions in Eq 2.

The membrane temperature field $\theta_{C}(x,t)$ is governed by

$$\frac{\partial \theta_{C}(x,t)}{\partial t}=\frac{\nabla \cdot \left(\kappa_{CC}\nabla \theta_{C}(x,t)\right)}{c_{C}}-\frac{\kappa_{CI}(x;X)\left(\theta_{C}(x,t)-\theta_{I}\right)}{c_{C}}\;-\frac{c_{0}\nabla \Phi :\bar{\kappa }\nabla q(x,t)}{c_{C}}+\frac{\left(c_{0}\nabla \Phi \right):\frac{1}{\gamma }\left(c_{0}\nabla \Phi \right)}{c_{C}}+{𝐠}_{thm,\theta_{C}}(x,t).$$ (3)

The thermal conductivity of the membrane and interfacial region is denoted by $\kappa_{CC}$, $\kappa_{CI}$ and the specific heat of the membrane is denoted by *C*_*C*_. The fluctuations are given by ${𝐠}_{thm,\theta_{C}}$. We model the spatial fields of the system as having periodic boundary conditions in Eq 3.

**Fig 1:**
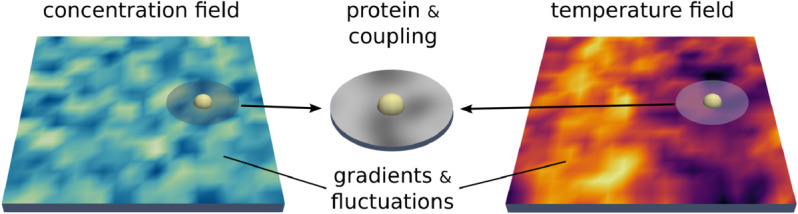
Membrane-protein drift-diffusion: Stochastic non-equilibrium model. Shows how the protein drift-diffusion dynamics are coupled to surrounding concentration and temperature fields. The interfacial region of surrounding lipids is shown as a circular patch to denote the coarse-grained range over which the coupling is active. This is modeled through the term $\kappa_{CI}(x,X)$ in Eqs 3 and 4.

We model the nearby region of lipids surrounding the protein and its surface as an interfacial region with temperature $\theta_{I}$. This models the coarse-grained coupling between a protein and lipids allowing for modeling effects that are under-resolved in point-particle models. This allows for modeling additional state information for exchanges in energy and momentum between the protein and the membrane. We use in practice $\kappa_{CI}(x,X)=\kappa \eta (x-X)$ where η(r) is a radial function, such as a truncated Gaussian or Peskin *δ*-function [47,48]. We show the interfacial region and coupling in Fig 1.

The other temperatures are governed by

$$\frac{\partial \theta_{P}}{\partial t}=-\frac{\kappa_{PI}\left(\theta_{P}-\theta_{I}\right)}{c_{P}}+\frac{{𝐅}_{X}^{T}{𝐌}_{XX}{𝐅}_{X}}{c_{P}}+{𝐆}_{thm,\theta_{P}},\;\frac{\partial \theta_{I}}{\partial t}=\frac{\kappa_{PI}\left(\theta_{P}-\theta_{I}\right)}{c_{I}}+\int \frac{\kappa_{CI}(x;X)\left(\theta_{C}(x,t)-\theta_{I}\right)}{c_{I}}dx+{𝐆}_{thm,\theta_{I}}.$$ (4)

The specific heats of the protein and interface are denoted by *C*_*P*_, *C*_*I*_. The thermal conductivities are denoted by $\kappa_{PI}$, $\kappa_{CI}$. The fluctuations are given by ${𝐆}_{thm,\theta_{P}}$, ${𝐆}_{thm,\theta_{I}}$.

We use a force acting on the protein that is coupled to the local concentration field given by

$${𝐅}_{X}=-\partial_{X}U=-\nabla_{𝐗}\Psi (𝐗)+\int -\nabla_{𝐗}\Phi (x;𝐗)c_{0}q(x,t)dx.$$ (5)

The *q*(*x*,*t*) gives the fluctuating distribution of the concentration field of the chemical species that interacts with the protein [48,53,64]. The Ψ(𝐗) gives the potential energy of the protein. The Φ(r,𝐗) denotes a chemical potential for the free energy of the molecules of the chemical species *q*(*x*,*t*) to be at location *r*. We treat the system in an over-damped regime where the hydrodynamic flow that would be induced by the drag of the dilute chemical species is negligible for the concentration field. As seen in our model equations, the free energy that is associated with the force ${𝐅}_{X}$ and −∇Φ drives both the protein dynamics and fluxes of the concentration field of the chemical species.

The system temperatures *θ* and related quantities are denoted using notation of the form $\theta_{\left(\cdot \right)}$, with $\theta_{P}$ for the protein, $\theta_{C}$ for the membrane, and $\theta_{I}$ for the interfacial region. The thermal conductivities for heat exchanges are given by $\kappa_{\left(\cdot \right)}$ with $\kappa_{CC}$ for the membrane temperature field, $\kappa_{CI}$ between the interface and membrane field, and $\kappa_{PI}$ between the protein and interface. In this work, we also simplify the models by treating the hydrodynamic contributions of the membrane in the over-damped regime through the mobility tensor *M*_*XX*_ for the protein. This helps to mitigate sources of stiffness in the dynamics from the hydrodynamic relaxation time-scales. For some phenomena, this may yield results that differ from models that include the momentum of the hydrodynamic flows as in [51,53,60].

To account for the fluctuations we use Gaussian random fields ${𝐆}_{thm,*},{𝐠}_{thm,*}$. We discuss how to derive the specific form for these stochastic driving fields in the next section. We denote the vector-valued terms as ${𝐆}_{thm}(t)$ and the stochastic spatial fields as ${𝐠}_{thm}(x,t)$. To obtain these terms requires a non-equilibrium statistical mechanics analysis of our system.

### Non-equilibrium statistical mechanics of the protein-membrane system

To provide a unified approach for analyzing the system to derive the fluctuations and to develop our simulation methods, we also provide a more abstract reformulation of our model given in Eqs 1–4. We express our model using stochastic dynamics of the form

$$\frac{d𝐘}{dt}={\bar{K}}^{\left(1\right)}𝒟𝒮+{\bar{K}}^{\left(2\right)}𝒟𝒮+{\bar{K}}^{\left(3\right)}𝒟𝒮+{𝐠}_{thm},$$ (6)

Our formulation is based on the non-equilibrium statistical mechanics framework GENERIC of Ottinger in [65,66]. We collect the system state variables together into the vector $𝐘(t)=[𝐗(t),q(t),\theta_{P}(t),\theta_{C}(t),\theta_{I}(t)]$. This allows for a uniform treatment of phenomena, including diffusivities that are dependent on location, temperature, or even friction that can have non-linear responses to velocity. This reformulation also provides insights helpful in analysis and derivations for obtaining the appropriate fluctuations and for developing numerical approximations. We perform analysis and develop stochastic numerical methods that help ensure null-space alignment between the operators of the reversible and irreversible processes and preserve other properties of the dynamics.

The energy of the system is denoted by ℰ, the entropy is denote by $𝒮=S^{\left(1\right)}\;+\;S^{\left(2\right)}\;+\;S^{\left(3\right)}$. The entropy *S*^(1)^ is associated with the protein, *S*^(2)^ with the membrane, *S*^(3)^ with the interfacial region. The gradient in 𝐘 of the entropy is denote by 𝒟𝒮. The dissipative irreversible processes in the dynamics are modeled by the collection of symmetric positive definite operators ${\bar{K}}^{\left(j\right)}$. We give more details of the entropy and these operators for our model below and in S1 Appendix.

We model the fluctuations as ${𝐠}_{thm}={𝐠}_{thm}^{\left(1\right)}+{𝐠}_{thm}^{\left(2\right)}+{𝐠}_{thm}^{\left(3\right)}$ with

$${𝐠}_{thm}^{\left(j\right)}=k_{B}(\nabla \cdot K^{\left(j\right)})+B^{\left(j\right)}\frac{dW_{t}^{\left(j\right)}}{dt},$$ (7)

where $B^{\left(j\right)}B^{(j),T}=2k_{B}K^{\left(j\right)}$. The $dW_{t}^{\left(j\right)}$ are increments of the Wiener process independent in *j* [67,68]. The *k*_*B*_ is the Boltzmann constant [63]. We obtain the correlations for the stochastic driving fields ${𝐠}_{thm}^{\left(j\right)}$ by combining the increments $dW_{t}^{\left(j\right)}$ using the operators *B*^(*j*)^. We use Eq 7 to obtain the fluctuations for our system, from the dissipative operators *K*^(*j*)^. We discuss these operators for our model below.

For our model in Eq 1–4, the energy is given by

$$ℰ(𝐘)={ℰ}^{\left(1\right)}(𝐘)+{ℰ}^{\left(2\right)}(𝐘)+{ℰ}^{\left(3\right)}(𝐘).$$ (8)

The particle energy is given by ${ℰ}^{\left(1\right)}(𝐘)=\Psi (𝐗)+c_{P}\theta_{P}.$ The potential energy is denoted by Ψ(𝐗). The energy of the concentration field depends on a spatial chemical potential Φ(x;𝐗) with

$${ℰ}^{\left(2\right)}(𝐘)=\int \Phi (x;𝐗)c_{0}q(x)dx+\int c_{C}\theta_{C}(x)dV_{x}.$$ (9)

The interface has the thermal energy ${ℰ}^{\left(3\right)}(𝐘)=c_{I}\theta_{I}.$ We consider entropy arising from the particle, membrane, and interfacial coupling

$$𝒮(𝐘)={𝒮}^{\left(1\right)}+{𝒮}^{\left(2\right)}+{𝒮}^{\left(3\right)}.$$ (10)

The entropy associated with the particle temperature $\theta_{P}$ is

$${𝒮}^{\left(1\right)}=c_{P}\ln(\theta_{P}).$$ (11)

The membrane concentration field *q*(*x*) and temperature $\theta_{C}(x)$ contribute the entropy

$${𝒮}^{\left(2\right)}=𝒮(q,\theta )=-\int c_{0}q(x)\ln(q(x))dx+\int c_{C}\ln(\theta_{C}(x))dV_{x}.$$ (12)

Entropy also arises from the temperature $\theta_{I}$ we track at the exchanges at the interface between the particle and the membrane

$${𝒮}^{\left(3\right)}=c_{I}\ln(\theta_{I}).$$ (13)

We express the entropy gradient as

$$𝒟𝒮={\left[\partial_{𝐗}𝒮,\partial_{q(x)}𝒮,\partial_{\theta_{P}}𝒮,\partial_{\theta_{I}}𝒮,\partial_{\theta_{C}(x)}𝒮\right]}^{T}.$$ (14)

This has the contributions

$$\begin{array}{cccccccc} \partial_{𝐗}𝒮 & = & 0, & \partial_{q(x)}𝒮 & = & -c_{0}\left(1+\ln(q(x))\right), & & \\ \partial_{\theta_{P}}𝒮 & = & c_{P}/\theta_{P}, & \partial_{\theta_{I}}𝒮 & = & c_{I}/\theta_{I},\\ \partial_{\theta_{C}(x)}𝒮 & = & c_{C}/\theta_{C}(x). \end{array}$$ (15)

The energy gradient can be expressed as

$$𝒟ℰ={\left[\partial_{𝐗}ℰ,\partial_{q(x)}ℰ,\partial_{\theta_{P}}ℰ,\partial_{\theta_{I}}ℰ,\partial_{\theta_{C}(x)}ℰ\right]}^{T}.$$ (16)

This has components

$$\begin{array}{cccccc} \partial_{𝐗}ℰ & = & \nabla_{𝐗}\Psi (𝐗)+\int \nabla_{𝐗}\Phi (x;𝐗)c_{0}q(x)dx, & \partial_{q(x)}ℰ & = & c_{0}\Phi (x;𝐗),\\ \partial_{\theta_{P}}ℰ & = & c_{P}, & \partial_{\theta_{I}}ℰ & = & c_{I},\\ \partial_{\theta_{C}(x)}ℰ & = & c_{C}. \end{array}$$ (17)

This provides a statistical mechanics analysis of our model in Eq 1 and a systematic way to derive the associated fluctuations and stochastic driving fields. In particular, by expressing the dynamics in terms of ${\bar{K}}^{\left(1\right)}$, ${\bar{K}}^{\left(2\right)}$, and ${\bar{K}}^{\left(3\right)}$ for Eqs 1–3, we can use Eq 7 to obtain the stochastic driving fields ${𝐠}_{thm}^{\left(1\right)}$, ${𝐠}_{thm}^{\left(2\right)}$, and ${𝐠}_{thm}^{\left(3\right)}$. We give the operators *K*^(*j*)^ for our model in Eq 1 in S1 Appendix. This provides the form of the fluctuation terms for the membrane-protein system dynamics in Eqs 1–3. For performing practical simulations of the protein-membrane system, numerical methods are required to discretize the equations and to generate efficiently the samples of the stochastic terms ${𝐠}_{thm}^{\left(j\right)}$.

### Stochastic numerical methods for simulations of the membrane-protein system

We now discuss briefly our simulation approaches and stochastic numerical methods for capturing the discrete particle drift-diffusion dynamics and the fluctuations of the continuum concentration and temperature fields. We remark that we focus in this paper primarily on the biophysical motivations of the work and will discuss further technical details of the developed numerical methods elsewhere. We provide further details on the methods in S2 Appendix and numerical validation studies for convergence in S3 Appendix.

#### Temporal discretization and time-step integration.

We discretize and integrate the stochastic dynamics in Eq 6 using the following two-stage approach

$${\tilde{Y}}^{n+1}=Y^{n}+a(Y^{n})\Delta t+\sum_{j}b^{\left(j\right)}(Y^{n})\Delta W^{n,j}\;Y^{n+1}=Y^{n}+\frac{1}{2}\left(a(Y^{n})+a({\tilde{Y}}^{n+1})\right)\Delta t+\frac{1}{2}\sum_{j}\left(b^{\left(j\right)}(Y^{n})+b^{\left(j\right)}({\tilde{Y}}^{n+1})\right)\Delta W^{n,(j)}.$$ (18)

The $a(Y)=L(Y)\nabla E(Y)\;+\;\sum_{j}\nabla K^{\left(j\right)}(Y)S^{\left(j\right)}(Y)$ and $b^{\left(j\right)}(Y)=B^{\left(j\right)}(Y)$. The Wiener increments $\Delta W^{n,(j)}$ denote Gaussian random variates having mean zero and variance $\langle \Delta W^{n_{1},j_{1}}\Delta W^{n_{2},j_{2}}\rangle =\delta_{n_{1},n_{2}}\delta_{j_{1},j_{2}}\Delta t$. Our integrator is based on a variant of the Euler-Heun Method [69]. There also have been alternative integrators developed for related non-equilibrium formulations in [66]. An important part of the updates is that the same increments $\Delta W^{n,j}$ are used in both steps. This serves as part of how the contributions of the divergence term are handled implicitly by the numerical methods [69]. We remark that the updates are equivalent to approximating the Stratonovich formulation of the stochastic process where fluctuations are treated as $g_{thm}^{\left(j\right)}=B^{\left(j\right)}\;\circ \;dW_{t}^{\left(j\right)}$. When giving the process the Ito interpretation the ∘ operator would expand the expression to include the drift divergence term as above. The *B*^(*j*)^ satisfy $B^{\left(j\right)}B^{(j),T}=2k_{B}K^{\left(j\right)}$ with the Boltzmann constant *k*_*B*_ and the operators *K*^(*j*)^ given above.

#### Spatial discretization of the continuum fields.

**Fig 2:**
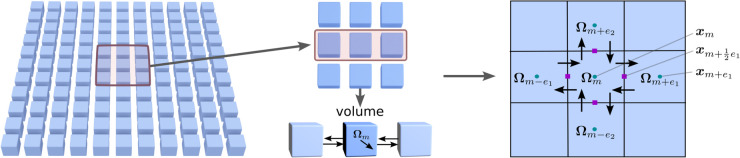
Spatial discretization. The system is spatially discretized using a finite volume approach where continuum fields on $\Omega =\cup_{m}\Omega_{m}$ are divided into a finite collection of volumes $\Omega_{m}$. The gradients and divergences are approximated by discrete operators 𝒢 and 𝒟 modeling the fluxes and exchanges between the volumes. This ensures the stochastic numerical methods adhere to physical conservation and adjoint conditions.

We spatially discretize the system using a finite volume approach. This is done by providing discretizations for the divergence 𝒟 and gradient 𝒢. We generate numerical methods for the spatial discretization by replacing throughout in our analytic expressions ∇ by the discrete operator 𝒢 and ∇·=div by discrete operator 𝒟. We build on our finite volume methods in [47,64]. Recently, there also has also been work on discretization methods using finite elements and other discretizations to generate fluctuations with *O*(*N*) complexity in [49,50]. In our finite volume methods, we replace spatial integrals ∫(·)dx by the corresponding finite sums $\sum_{m}(\cdot {)}_{x_{m}}\delta V$. We treat continuum bodies as divided into a discrete finite collection of boxes each having volume δV. The *x*_*m*_ denotes the location of the *m*^*th*^ finite volume box, see Fig 2.

We consider fields represented by average values at the volume centers ${𝐱}_{m}$. We discretize the operators by considering fluxes 𝐉 at location of the volume boundaries ${𝐱}_{m\pm \frac{1}{2}e_{d}}$, using related conventions as in our finite volume methods in [47,64]. The *e*_*d*_ denotes the standard basis vector with zeros for all entries except for the value 1 for entry with index *d*. This is achieved by discretizing each of the gradient operators 𝒢=grad(·) of the field values 𝐅 with components *F*^(*d*)^ by using the central differences

$$\text{grad}(𝐅{)}^{\left(d\right)}({𝐱}_{m\pm \frac{1}{2}e_{d}})=𝒢(𝐅{)}^{\left(d\right)}({𝐱}_{m\pm \frac{1}{2}e_{d}})=\pm \frac{1}{\Delta x}\left(F^{\left(d\right)}({𝐱}_{m\pm e_{d}})-F^{\left(d\right)}({𝐱}_{m})\right).$$ (19)

The fluxes are given by ${𝐉}^{\left(d\right)}({𝐱}_{m\pm \frac{1}{2}e_{d}})=\text{grad}(𝐅{)}^{\left(d\right)}({𝐱}_{m\pm \frac{1}{2}e_{d}})$ We discretize the divergence operators 𝒟=div(·) of the fluxes by the central differences

$$\text{div}(𝐉)({𝐱}_{m})=𝒟(𝐉)({𝐱}_{m})=\frac{1}{\Delta x}\sum_{d=1}^{n}\left[{𝐉}^{\left(d\right)}({𝐱}_{m+\frac{1}{2}e_{d}})-{𝐉}^{\left(d\right)}({𝐱}_{m-\frac{1}{2}e_{d}})\right].$$ (20)

This corresponds to discretizing within the operator *K*^(*j*)^ the gradient and divergence operators using 𝒢 and 𝒟 above in Eqs 1–4. We further have that the discretized gradient operator 𝒢 appearing in these expressions is the negative adjoint of the discretized divergence operator 𝒟, so $𝒢=-{𝒟}^{T}$. Our finite volume approach provides a systematic way to obtain discretizations and stochastic numerical methods that satisfy properties such as the adjoint relations between gradient and divergence which help preserve structural features of the dynamics important in their statistical mechanics [52,64]. This provides numerical methods for handling the spatial discretization and time-step integration of our model in Eq 1–4.

#### Methods for generating fluctuations.

To obtain practical simulation methods, we need to handle the fluctuations of the continuum concentration and temperature fields of the membrane given by ${𝐠}_{thm}^{\left(j\right)}$ in Eq 7. This requires being able to generate efficiently the stochastic driving fields each time-step ${𝐡}_{thm}^{(j),n}=B^{\left(j\right)}(Y)\Delta W^{n,j}$, where $B^{\left(j\right)}B^{(j),T}=2k_{B}K^{\left(j\right)}$. Methods such as Cholesky Factorizations are prohibitive for the continuum fields given that they scale as *O*(*N*^3^) in the number of degrees of freedom *N* [52,70]. Further, the operator $B^{\left(j\right)}=B^{\left(j\right)}({𝐘}^{n})$ in general depends on the state ${𝐘}^{n}$ which would require recomputing these factors each time-step as the state changes. We show alternatives can be developed avoiding these issues through a combination of analytic factorizations and further reductions. We generate the fields using the formulation

$${𝐡}_{thm}^{\left(j\right)}=\sqrt{\Delta t}B^{\left(j\right)}\xi^{\left(j\right)}=\sqrt{2k_{B}\Delta t}R^{\left(j\right)}\xi^{\left(j\right)},$$ (21)

where the $\xi^{\left(j\right)}∼\eta (0,I)$ are standard Gaussian random variates and *R*^(*j*)^ is a factor satisfying $K^{\left(j\right)}=R^{\left(j\right)}R^{(j),T}$. We perform analysis to find explicit expressions for the factors *R*^(*j*)^ for the proteins, interface, and membrane in S2 Appendix. This allows us to generate the needed stochastic fields efficiently each time step needed in Eqs 21 and 7. We decompose the operators into parts allowing for generation with sampling methods having computational complexity at most *O*(*N*^2^). For some terms we are able to obtain better results by using sparsity and other structures to achieve algorithms having sampling complexity *O*(*N*). We give further details on our analytic factorizations and discussion of our stochastic sampling methods in S2 Appendix and S3 Appendix.

## Results

We investigate heterogeneous membranes and the impacts of spatial variations in concentration and temperature on the drift-diffusion dynamics of proteins. We consider (i) how concentration gradients and kinetics can drive the spatial organization of proteins, (ii) the roles of fluctuations in the encoding of signals by proteins to sense external thermal gradients, and (iii) how localized laser heating can be used to probe protein escape kinetics from local energy wells within heterogeneous membranes. The results demonstrate a few ways the simulation approaches can be used to study biological mechanisms within membranes and related non-equilibrium phenomena in other systems.

### Protein drift-diffusion in concentration gradients of heterogeneous membranes

Protein organization can be governed by other cellular metabolic activities that create regions with enhanced concentrations of chemical species [2,3,6]. We model the concentration of a signaling chemical species by the field *q*(*x*). We consider the case where there is an interaction energy of the form

$$𝒱(𝐗;q)=\int \eta (x-𝐗)c_{0}q(x)dx+\Psi (𝐗).$$ (22)

This has the associated force

$${𝐅}_{X}=-\nabla_{X}𝒱(𝐗;q)=\int \nabla_{x}\eta (x-𝐗)c_{0}q(x)dx-\nabla_{X}\Psi (𝐗).$$ (23)

We use for the coupling kernel

$$\eta (|s|)=\frac{k_{1}}{Z}\exp\left(\frac{-|s{|}^{2}}{2\sigma_{0}^{2}}\right),\;Z={\left(2\pi \sigma_{0}^{2}\right)}^{d/2}.$$ (24)

For the membrane, we take *d* = 2, *k*_1_ = 1.1 for the coupling strength, and $\sigma_{0}=0.2$. The energy in Eq 22 is motivated by the free-energy associated with interactions between the signaling molecules and the protein, such as through electrostatics or other physical effects. As a basic model we use the coupling kernel in Eq 24 to give a model for a localized decaying interaction.

As an initial model for a single particle, we use for simplicity the mobility ${𝐌}_{XX}=(1/\gamma_{p})ℐ$. The $\gamma_{p}$ denotes the effective hydrodynamic drag of the particle for translational motions within the membrane. More sophisticated models for multiple particles can also be formulated using the Saffman-Delbruck theory and other hydrodynamic approaches, see [48,51,71]. Using these models our approaches readily can be extended to take into account the hydrodynamic interactions mediated by flow of the surrounding lipids of the membrane and the solvent.

**Fig 3:**
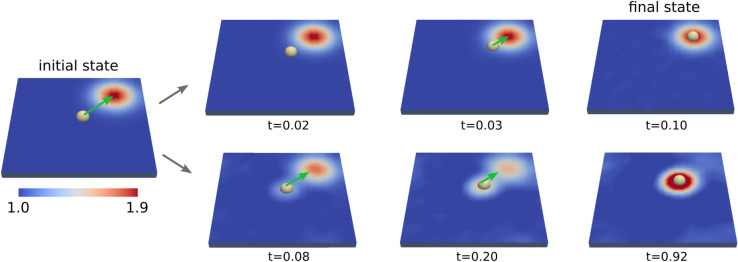
Protein positioning through concentration of signaling molecules. We show how an individual protein interacts with the concentration field of signaling molecules with the affinity given in Eq 22. We show the evolution over time *t* with the parameters in Table 1. Depending on the relative time-scales of the signaling molecule diffusivity and protein dynamic time-scale, there are different behaviors. When the signaling molecule diffusivity is small, the protein localizes to the initial concentration location *(top)*. When the signaling molecule diffusivity is larger, the protein does not move significantly and the concentration collects around the initial location of the protein *(bottom)*.

In our simulation studies, we consider the case where there is initial concentration of a signaling chemical species in a localized region centered at *x*_1_. This is modeled by a Gaussian concentration with mean $\mu_{0}=x_{1}=[1.5,1.5]$ and variance $\sigma^{2}$ with σ=0.2. We then consider how the simultaneous concentration drift-diffusion of *q*(*x*,*t*) over time interacts with the particle drift-diffusion 𝐗(t) when $𝐗(0)=x_{0}=[0,0]$. Given the affinity between the particle and signaling chemical species, we see over time they will occupy the same location through mutual attraction, see Fig 3. On much longer time-scales the particle and signaling chemical species can also diffuse together throughout the domain. We investigate the localization of the protein and signaling species concentration field when varying the protein drag $\gamma_{p}$ and the signaling species diffusivity $\bar{\kappa }$. We show a few cases for how the particle and concentration field evolve in Fig 3.

**Fig 4:**
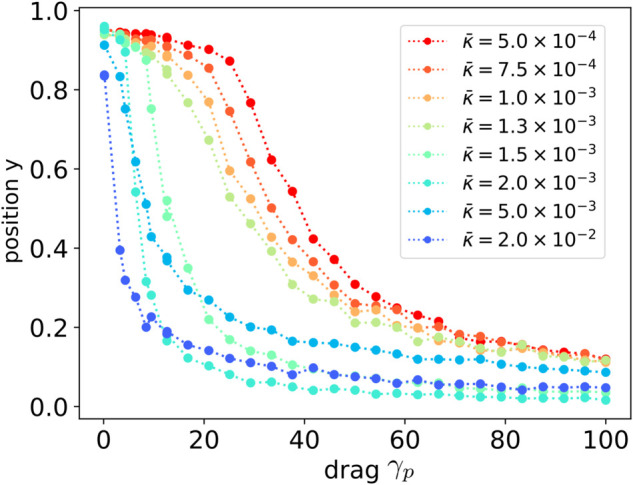
Protein positioning through concentration of signaling molecules. We show how key factors impact the positioning of proteins in response to attraction to a region of large concentration of a signaling molecule. The scaled final protein position $y=(x^{\left(2\right)}\;-\;x_{0}^{\left(2\right)})/(x_{1}^{\left(2\right)}-x_{0}^{\left(2\right)})$ is shown in range 0.0 and 1.0. This gives the fraction of the distance it moved toward the signaling molecule initial source location *x*_1_. We consider cases when varying the signaling molecule diffusivities $\bar{\kappa }$ and the effective hydrodynamic radius of the protein characterized by the viscous drag $\gamma_{p}$. The localization of the protein involves a competition between the diffusion of the signaling molecules and protein motion toward regions of larger concentration. We also show a few example cases in Fig 3.

We remark that throughout, we consider non-dimensional treatment of the equations. For physical intuition and connecting our results to particular biological systems, the results of the simulation methods can be converted to dimensional units by taking a collection of reference scales. In particular, for length $\bar{\ell }$, mass $\bar{m}$, time $\bar{t}$, and temperature $\bar{T}$. For example, we can take as our characteristic reference scales $\bar{\ell }=10$ nm, $\bar{m}=50$ kDa for kilodalton units (kDa), $\bar{t}=1$ *μ*s, and $k_{B}\bar{T}=4.1\;\times \;10^{-21}$ J. For a given non-dimensional quantity *z*, there is a characteristic scale $\bar{Z}$ so that for the dimensional quantity $\tilde{z}$ we have non-dimensionalization $z=\tilde{z}/\bar{Z}$. We can recover the corresponding dimensional quantity as $\tilde{z}=z\bar{Z}$. We give non-dimensional parameters for simulations of our system in Table 1. We show the results in Fig 4.

**Table 1:** Parameters for the concentration gradient model. We give the values for the SELM simulations of the signaling molecule concentration fields and particle drift-diffusion dynamics.

parameter		value	parameter		value
$\bar{\kappa }$	concentration diffusion	$1.2\times 10^{-3}$	*C*_*P*_	specific heat: particle	1.2
*c*_0_	total concentration	2.1	*C*_*C*_	specific heat: membrane	$1.3\times 10^{2}$
$\kappa_{PI}$	heat conduction: particle	$8.2\times 10^{6}$	*C*_*I*_	specific heat: interface	$1.4\times 10^{2}$
$\kappa_{CI}$	heat conduction: interface	$3.0\times 10^{3}$	$\gamma_{p}$	particle drag	1.3 × 10^1^
$\kappa_{CC}$	heat conduction: membrane	$1.3\times 10^{2}$	$\theta_{0}$	baseline membrane temperature	3.0
$\kappa_{0}$	heat conduction: fluid	$2.1\times 10^{-3}$	*k*_*B*_	Boltzmann’s constant	1.0 × 10^−5^
*n*_*x*_	number grid points in x	2.0 × 10^1^	Δx	mesh spacing	1.0 × 10^−1^
*n*_*y*_	number grid points in y	2.0 × 10^1^	Δt	time step	1.0 × 10^−3^

We find that varying the signaling molecule diffusivity $\bar{\kappa }=\theta_{C}/\gamma$ and the protein drag $\gamma_{p}$ can be used to regulate localization. The final location of the protein depends on the ratio of the time-scale $\tau_{s}=\ell^{2}/\bar{\kappa }$ for the signaling chemical species to diffuse and the time-scale $\tau_{p}=\ell \gamma_{p}/f_{0}$ for the protein motion. The ℓ is the initial separation distance and *f*_0_ the strength of the initial interaction force on the protein. If $\tau_{s}$ is small relative to $\tau_{p}$, then we find the protein moves to location *x*_1_ of the initial large concentration. If the $\tau_{s}$ is large relative to $\tau_{p}$, we find the signaling species migrates to surround the protein at location *x*_0_ before the protein has the chance to move significantly.

At intermediate time-scales we find there is a combination of these effects with both the signaling molecules and protein meeting at a location in-between the locations *x*_0_ and *x*_1_. Interestingly, as the diffusivity of the signaling molecule becomes very large there can be some reversals since it can accumulate rapidly as it moves all at once toward the protein which results in a large localized force that briefly pulls the protein toward the location *x*_1_. We show the results of our studies in Fig 4.

### Thermal gradient sensing and fluctuations

**Fig 5:**
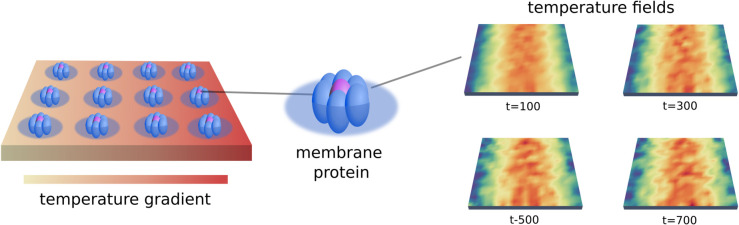
Sensing of thermal gradients. We consider the responses of thermal sensitive proteins and how they may encode information about spatial temperature variations obscured by fluctuations *(left)*. We show SELM simulations of fluctuating temperature fields having an initial periodic structure given by $\theta_{C}(𝐱,0)=\theta_{0}\left(1.0+a_{0}\sin(\pi 𝐤\cdot 𝐱/L)\right)$ with 𝐤=[1,0] and amplitude *a*_0_ = 1.0, *(right)*.

The detection of changes in temperature and thermal gradients plays an important role in many types of cells. This includes intracellular processes involved in modulating growth [72–74] and for single cell micro-organisms the ability to control migration toward or away from heat sources [75–77]. We consider thermal sensitive proteins, such as the channel proteins TRP that have temperature dependent gating dynamics [78–80] for detecting changes [72,77,81]. In our modeling, these are patterned within the membrane at fixed locations and we investigate how they could encode spatial variations of the temperature when obscured by fluctuations, see Fig 5.

For the transmission of heat to the local protein from the area of the surrounding temperature field, we use

$$\bar{Q}({𝐱}_{i},t)=\int \zeta (𝐱-{𝐱}_{i})\theta_{C}(𝐱,t)dx,\;\;\zeta (|s|)=\frac{1}{Z}\exp\left(\frac{-|s{|}^{2}}{2\sigma_{0}^{2}}\right),\;\;Z={\left(2\pi \sigma_{0}^{2}\right)}^{d/2}.$$ (25)

This $\bar{Q}$ models the temperature that would be sensed by a fixed protein, such as a TRP channel at location ${𝐱}_{i}$ within the membrane [79]. We use parameters *d* = 2 and $\sigma_{0}=0.1$. We investigate the fidelity by which the proteins can collectively be used to sense spatial variations in the temperature in the presence of fluctuations. Since TRP channel gating gives a permeability to ions that depends on the local temperature, this can be used in chemical reactions to increase the local concentration of activated protein molecules. In this way the spatial variations in the concentration of the activated protein species $I({𝐱}_{i},t)$ can be used to locally encode the external temperature gradient for use in further downstream chemical reactions within the cell. We can consider reactions similar to those that arise in chemotaxis, such as the models in [64,82].

For chemical reactions with small spatial diffusion having motifs that arise in chemotaxis, the final concentrations that rapidly reach their equilibrium can be obtained by a reduction to a time-averaged response function of the input signal [64,83,84]. The equilibration of the chemical reactions serves to produce a signaling filter for the gradient and fluctuations. As a model for such a reduction, we use an effective time-averaged signal encoded in the local concentration $\bar{I}$. We use the averaging

$$\bar{I}({𝐱}_{i},t)=\beta_{0}\int_{-\infty }^{t}\eta (t-s)\bar{Q}({𝐱}_{i},s)ds.$$ (26)

The *η* gives the response function given the local reaction chemistry. Motivated by first-order chemical reactions for activation and deactivation of a protein species for encoding, we consider exponential response functions. In particular, we use the response function η(τ)=λexp(−λτ) with $\lambda =10^{4}$, $\beta_{0}=1/3$. Other chemical reaction motifs would correspond to different choices of the response function *η* [64,82,83,85].

The $\bar{I}$ gives the concentration of the signaling molecules that encode the spatially-temporally filtered temperature signal. The signaling concentrations $\bar{I}({𝐱}_{i})$ can be further coupled to downstream chemical reactions that impact cellular processes [64,82,85]. We focus here on this initial processing of signals from the surrounding fluctuating temperature fields.

**Fig 6:**
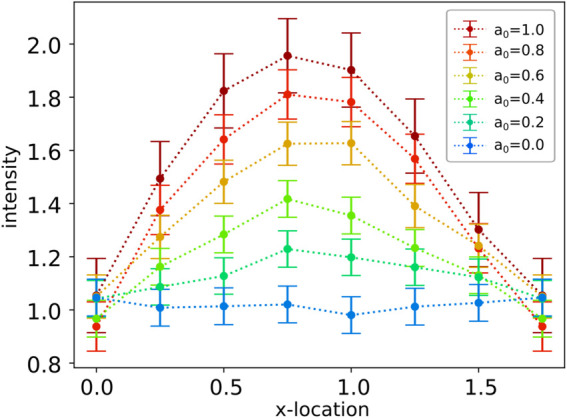
Sensing thermal gradients. We show results for protein responses for encoding signals from a spatially varying temperature field subject to fluctuations. Shown is the average intensity of the indicator $\bar{I}$ concentration and one standard deviation as error bars. The *x* is the location along the membrane and ranges over the width of the simulation domain $L=n_{x}\Delta x$. We investigate how the sensed signals change as the amplitude *a*_0_ of the spatial temperature fields are varied.

We investigate the thermal sensing as the amplitude of the spatial variations of the external temperature field is varied. We consider the case of spatial variations that start from an initial temperature profile $\theta_{C}(𝐱)=\theta_{0}\left(1+a_{0}\sin(\pi 𝐤\cdot 𝐱/L)\right)$, with 𝐤=[1,0] and *a*_0_ = 1.0. The parameters of our model and simulation studies are given in Table 2. We show results in Fig 6.

**Table 2:** Parameters for the temperature sensing model. We give the values for the SELM simulations of protein sensing of fluctuating temperature variations.

parameter		value	parameter		value
$\kappa_{PI}$	heat conduction: particle	$8.2\times 10^{6}$	*C*_*P*_	specific heat: particle	1.0
$\kappa_{CI}$	heat conduction: interface	0.0	*C*_*C*_	specific heat: concentration	4.0 × 10^1^
$\kappa_{CC}$	heat conduction: membrane	$8.2\times 10^{4}$	*C*_*I*_	specific heat: interface	$1.4\times 10^{2}$
$\kappa_{0}$	heat conduction: fluid	$8.2\times 10^{4}$	$\theta_{0}$	baseline membrane temperature	3.0
*c*_0_	total concentration	2.1	*k*_*B*_	Boltzmann’s constant	1.0 × 10^−3^
*n*_*x*_	number grid points in x	2.0 × 10^1^	Δx	mesh spacing	1.0 × 10^−1^
*n*_*y*_	number grid points in y	2.0 × 10^1^	Δt	time step	1.0 × 10^−5^

We find while fluctuations can obscure significantly temperature gradients on small spatial-temporal scales this can be mitigated by processes that serve to filter the signal. In our simulations the temperature fields start with an initial sinusoidal profile and evolve over time toward a uniform equilibrium while also undergoing spontaneous fluctuations from transient local energy exchanges. We can see that for the largest amplitudes the signal of temperature changes can be detected, but becomes suppressed by the filtering over time and space as seen in the indicator species responses $\bar{I}$. For the smallest amplitudes, we see the gradient becomes obscured by noise. These simulations indicate some of the interesting trade-offs between the level of filtering to obtain a reliable signal while still resolving the spatial and temporal information inherent in the surrounding temperature fields relevant for biological responses, see Fig 6. This gives some demonstrations of how the non-equilibrium SELM simulation approaches can be utilized to investigate biophysical signal transduction of temperature gradients.

### Hot Brownian motion of particles in temperature gradients

We consider the non-equilibrium diffusion of particles that can undergo temperature changes from environmental and external heating. These thermal effects can drive more rapid particle diffusion and other phenomena referred to as Hot Brownian Motion [55,56]. Recent theoretical and simulation work studying these effects include [55–57,86]. In these studies, a description of the Brownian motion of a particle subject to laser heating is developed where temperature differences augment the local viscosity and fluctuations. Related experiments were also performed by laser heating gold nanoparticles that exhibit significant variations in their diffusion [56]. In other experimental observations, heating caused locally by catalytic enzymatic reactions also were found to impact diffusion [86].

The current theoretical studies reduce descriptions to particle-based models that use a separation of time-scales between the changes in the particle temperature and the spatial changes in the surrounding temperature fields of the environment. For systems that have more persistence or externally imposed spatial gradients, we show how our SELM modeling and simulation approaches can be used to capture further spatial-temporal effects. For example, the impacts of spatial heterogeneity, energy transfer and other augmentations from past locations that hot particles visit, or other time-scales associated with the surrounding environment.

We develop models capturing the ambient temperature field evolution and spatial gradients in conjunction with the temperature variations of the particles undergoing Brownian motion. As a specific system, we consider particles that can become transiently trapped within energy wells created by structures within the membrane. We investigate how the non-equilibrium particle diffusions impact the kinetics of escape from the energy wells. In our studies, the particles can diffuse into or out of different parts of the membrane that are subject to external optical heating. As the particles diffuse, they can heat up or cool down impacting their drift-diffusion dynamics in the heterogeneous energy landscape of the membrane. This impacts their escape kinetics from the energy wells.

We consider heterogeneous microstructures within the membrane that create energy wells of the form

$$\Psi (𝐗)=\sum_{i}-c_{2}\exp\left[-\frac{{\left(𝐗-{𝐗}_{i}\right)}^{2}}{2\sigma_{0}^{2}}\right].$$ (27)

**Fig 7:**
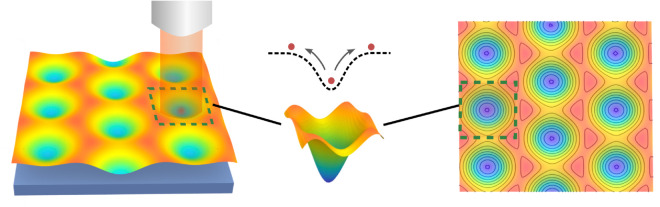
Hot Brownian motion in energy wells. We consider particles undergoing Brownian motions which can change temperature from energy exchanges with the surrounding environment. We study diffusion within a heterogeneous membrane where there are local energy wells some of which are heated by an external source. This impacts the particle diffusion within such wells and the time to escape by overcoming the energy barriers. We show results for particle escape times in Fig 8.

The ${𝐗}_{i}$ form a staggered lattice as shown in Fig 7. This generates particle forces

$${𝐅}_{X}=\sum_{i}-\nabla_{X}\Psi (𝐗)=-c_{2}\left(\frac{𝐗-{𝐗}_{i}}{\sigma_{0}^{2}}\right)\exp\left[-\frac{{\left(𝐗-{𝐗}_{i}\right)}^{2}}{2\sigma_{0}^{2}}\right].$$ (28)

These forces are taken to be generated by fixed microstructures of the membrane and the model does not involve the concentration field *q*. The protein is treated in the over-damped regime with mobility ${𝐌}_{XX}$ the same as in the previous models. We further consider external heating that creates a local region of elevated temperature within the membrane of the form

$$\theta_{m}(𝐱)=\theta_{0}\left(1+c_{3}\exp\left[-\frac{{\left(𝐱-{𝐱}_{0}\right)}^{2}}{2\sigma_{3}^{2}}\right]\right).$$ (29)

For example as induced by an external source laser source [37]. We illustrate the membrane-protein system in Fig 7.

We perform studies of a particle initially started in the center of an energy well at location ${𝐗}_{0}$. The initial temperature distribution in the membrane is non-uniform given by Eq 29. We consider the kinetics of Hot Brownian Motion and how the escape time is impacted by different particle temperature variations *c*_3_. In particular, we study the escape time for a particle to diffuse to radius *r*_0_ from the center ${𝐗}_{0}$ of the energy well. We perform simulations repeating this experiment for different strengths of the energy well *c*_2_ and for different amplitudes of temperature *c*_3_. The parameters used in our simulations are shown in Table 2.

We remark that the particle diffusivity is often characterized by the Mean Square Displacement (MSD), which is based on ensemble averaging of the particle’s motions over time. The diffusivity is then the derivative in time of the MSD. In the non-equilibrium setting, this statistic is less informative since it can exhibit more complicated non-linear behaviors on different time-scales as the particle moves in response to thermal gradient induced drifts, heats up or cools down, or diffuses to probe different parts of the membrane. As an alternative, we consider here the impact of thermal effects on the first-passage time statistics of the non-equilibrium system. The simulation methods for average well escape times we report can be used for those interested in estimating a renormalized effective diffusivity for protein behaviors over larger spatial-temporal scales [68,87].

**Table 3:** Parameters for hot Brownian motion. We give the values for the SELM simulations of the particle diffusing in heterogeneous temperature fields used in the studies for energy well escape kinetics.

parameter		value	parameter		value
$\kappa_{PI}$	heat conduction: particle	$5.7\times 10^{2}$	$\gamma_{p}$	particle drag	1.0 × 10^−1^
$\kappa_{CI}$	heat conduction: interface	$3.0\times 10^{3}$	*C*_*P*_	specific heat: particle	$9.3\times 10^{2}$
$\kappa_{CC}$	heat conduction: conc.	$2.1\times 10^{-3}$	*C*_*C*_	specific heat: membrane	$1.3\times 10^{4}$
$\kappa_{0}$	heat conduction: fluid	$8.2\times 10^{6}$	*C*_*I*_	specific heat: interface	$1.4\times 10^{2}$
*n*_*x*_	number grid points in x	2.0 × 10^1^	$\theta_{0}$	baseline membrane temperature	3.0
*n*_*y*_	number grid points in y	2.0 × 10^1^	*c*_2_	energy-well: strength	$1.5\times 10^{-4}$
Δx	mesh spacing	1.0 × 10^−1^	*c*_3_	external heating strength	(varies)
Δt	time step	3.0 × 10^−3^	*X*_0_	energy-well: center	[5.0/3.0,1.0]
*k*_*B*_	Boltzmann’s constant	1.0 × 10^−5^	-	-	-

**Fig 8:**
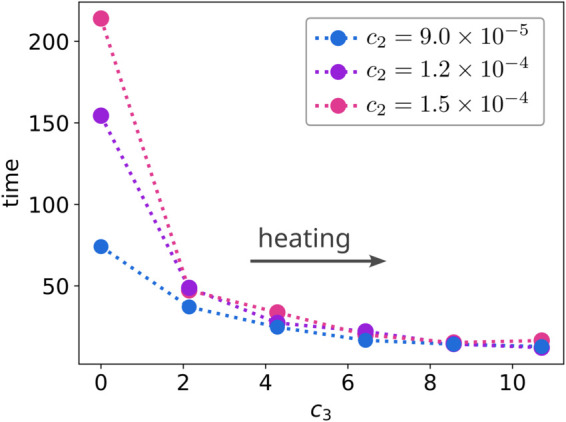
Hot Brownian motion in energy wells. We show the time for particles to escape from an energy well by diffusing to distance *r*_0_ from the well center. The membrane has a non-uniform temperature field modulated by *c*_3_ in Eq 29. This heats up the particles and can impact the energy well escape times. The *c*_2_ gives the strength of the energy well in Eq 27. We show how the particle escape times are impacted by the energy well strength and level of particle heating.

We show results in Fig 8. In the case of the strongest energy well $c_{2}=1.5\;\times \;10^{-4}$, we find at the baseline temperature $\theta_{0}=3.0$ with *c*_3_ = 0 the particle kinetics exhibit long-duration escape times. As the membrane is externally heated the particle temperature increases over time and the diffusive motions become larger and can more readily overcome the energy barriers. We see as *c*_3_ is increased these non-equilibrium diffusions have significantly smaller escape times than the baseline constant temperature case. When the external heating is large enough, the dominating time-scale becomes how long it takes for a particle to heat up beyond a critical temperature so *k*_*B*_*T* is a multiple of the energy barrier size, which allows for rapid escape. We see the escape times become negligible as we approach *c*_3_ = 10. We also see only a weak dependence on the energy well strength *c*_2_ as the external strength of heating *c*_3_ increases, see Fig 8.

These results show some of the ways the non-equilibrium SELM approaches can be used to capture phenomena in the drift-diffusion dynamics of particle kinetics within complex heterogeneous materials that have spatially varying microstructures and temperature variations over time. The results here indicate how trapped particles interacting with non-homogeneous temperature fields can impact kinetics. The SELM simulations also have the potential to capture interesting energy exchanges and augmentations where diffusing hot particles could locally heat up the membrane and change it locally which could impact kinetics in future encounters with previously visited locations. The temperature varying particles also provide mechanisms by which heat energy can be adsorbed and transferred to new locations and deposited through diffusion. The kinetics involved in such mechanisms involve an interplay between the rate of Brownian motion, which depends on the particle and local temperature fields, and the rates of particle heating and exchanges with the environment. The non-equilibrium SELM methods allow for capturing in a self-consistent manner such thermodynamics, kinetics, and related phenomena.

## Discussion

Biological systems involve active processes at the microstructure level that can drive membranes and proteins into interesting regimes that are out of thermodynamic equilibrium. Theoretical modeling frameworks and simulation methods were introduced for investigating non-equilibrium effects in proteins dynamics within heterogeneous membranes. The approaches are based on hybrid discrete-continuum descriptions which track discrete individual proteins and couple these to continuum fluctuating concentration and temperature fields. This allows for investigating the roles of non-equilibrium effects in the drift-diffusion dynamics of proteins and their coupling to spatial fields within the membrane associated with variations in concentration and temperature. Since the coupling is bi-directional, this also allows for studying exchanges of energy and other effects which impact the dynamical evolution of both the concentration and thermal spatial fields and the individual proteins.

The investigations show non-equilibrium effects can play a significant role impacting protein dynamics in mechanisms in biological systems and related *in vitro* experiments. It was shown that both variations in concentration of signaling molecules and their drift-diffusion kinetics can be used to regulate spatial localization of proteins within heterogeneous membrane structures. It was also shown that thermal effects can play a significant role within *in vitro* experiments for probing the drift-diffusion dynamics within the energy landscapes of heterogeneous membranes.

The introduced approaches provide self-consistent models for studying biophysical mechanisms involving the drift-diffusion dynamics of proteins within heterogeneous membranes in non-equilibrium regimes. The methods capture the energy exchanges between the mechanical and thermal parts of the system. It is expected these and related approaches can be used in studying diverse types of non-equilibrium phenomena involving mechanical-thermal coupling within biological systems and related *in vitro* experiments.

## Conclusion

We have developed theory and modeling approaches for investigating the non-equilibrium statistical mechanics of proteins immersed within heterogeneous membranes. We showed how these approaches could be used to obtain self-consistent models coupling the drift-diffusion dynamics of individual proteins with fluctuating continuum fields for concentration and temperature variations. We developed numerical methods for spatially discretizing the system and for efficiently generating the required stochastic driving fields accounting for the fluctuations for practical simulations. The resulting non-equilibrium approaches were used to investigate biological mechanisms for protein positioning and patterning within membranes, factors in thermal gradient sensing, and kinetics of Brownian motion of particles with temperature variations within energy landscapes of heterogeneous membranes. The approaches capture energy exchanges and fluctuations between the thermal and mechanical parts of the system allowing for investigating diverse non-equilibrium phenomena within biological systems and materials. This includes related applications in active soft materials, complex fluids, and other biophysical systems.

## Supporting information

S1 AppendixDiscussion of the irreversible operators *K*^(*j*)^ and the stochastic driving fields ${𝐠}^{\left(j\right)}$ of the membrane-protein system.(PDF)

S2 Appendix Discussion of the stochastic field generation methods and factorizations *R*^(*j*)^. (PDF)

S3 AppendixDiscussion of validation studies for the stochastic numerical methods.**Fig A in S3 Appendix. Transfer Operator Convergence.** We show how the transfer operator for the temperature field $\theta_{C}(x)$ converges as the spatial discretization Δx is refined. We test the accuracy of $\tilde{u}(𝐱,t)$ from the numerical methods at different time steps *t* using the predicted solution u(𝐱,t). We consider the maximum error over the grid. We find the numerical methods exhibit second-order convergence $O(\Delta x^{2})$ in agreement with theory. The spatial discretization Δx becomes smaller from left to right. The numerical tests were performed with default parameters in Table A in S3 Appendix.**Fig B in S3 Appendix. Covariance of Increments of the Stochastic Time-Step Integrator.** We show how the covariance of trajectories generated by the stochastic numerical methods compares with the target dynamics. Results are shown using a log scale. The numerical tests were performed with *n* = 10^4^ samples of the integration step with the parameters in Table A in S3 Appendix. We found a maximum absolute error of $\epsilon =6.8\times 10^{-9}$.**Table A in S3 Appendix. Parameters for the Stochastic Numerical Methods.** We give the default values used in tests.(PDF)
