# Supplementary material for: Protein drift-diffusion in membranes with non-equilibrium fluctuations arising from gradients in concentration or temperature
Source: PLoS Comput Biol. 2025 Nov 21;21(11):e1013678. doi: 10.1371/journal.pcbi.1013678 (PMC12654922; doi:10.1371/journal.pcbi.1013678)
Supplement: S1 Appendix — (PDF) [file pcbi.1013678.s001.pdf]

S1. Irreversible Operators $K^{(j)}$ and Stochastic Driving Fields $\mathbf{g}^{(j)}$ for Fluctuations of the Membrane-Protein System

For our protein-membrane model in equation 1, we can express the irreversible processes in the dynamics in terms of the dissipative operators $\bar{K}^{(j)}$. The protein drift-diffusion dynamics and temperature variations corresponds to

$$K^{(1)} = \begin{bmatrix} \theta_P \mathbf{M}_{XX} & -\frac{\theta_P \mathbf{M}_{XX} \nabla_{\mathbf{X}} \mathcal{E}}{c_P} \\ -\frac{\nabla_{\mathbf{X}} \mathcal{E}^T \theta_P \mathbf{M}_{XX}}{c_P} & \frac{\nabla_{\mathbf{X}} \mathcal{E}^T \theta_P \mathbf{M}_{XX} \nabla_{\mathbf{X}} \mathcal{E}}{c_P^2} \end{bmatrix} \begin{matrix} \mathbf{X} \\ \theta_P \end{matrix}. \quad \text{(columns: } \mathbf{X}, \ \theta_P\text{)} \tag{S1.1}$$

We have that $\mathbf{F}_X = -\nabla_X \mathcal{E} = -\partial_X U^T$. The concentration field diffusion and heat exchanges gives

$$K^{(2)} = \begin{bmatrix} -\text{div}\left(\frac{q(x)\bar{\kappa}}{c_0}\nabla\right) & \text{div}\left(\frac{q(x)\bar{\kappa}\Box c_0 \nabla\Phi(x)}{c_0 c_C}\right) \\ \frac{-c_0 \nabla\Phi(x):(q(x)\bar{\kappa}\nabla)}{c_0 c_C} & \frac{c_0 \nabla\Phi(x):(q(x)\bar{\kappa}\Box c_0 \nabla\Phi(x))}{c_0 c_C c_C} + \frac{-\nabla\cdot\left(\tilde{\kappa_0}\theta_C^2 \nabla\right)}{c_C} \end{bmatrix} \begin{matrix} q(x) \\ \theta_C \end{matrix}. \quad \text{(columns: } q(x), \ \theta_C\text{)} \tag{S1.2}$$

The $\Box$ denotes for the action of the operator acting on a spatial field where to substitute the input field, such as $q(x), \theta_C(x)$. We have that $-\delta_q \mathcal{E} = c_0 \Phi$ from equation 17. The interfacial coupling has heat exchanges that yield

$$K^{(3)} = \begin{bmatrix} \frac{\kappa_{PI}\theta_I\theta_P}{c_{P,P}} & 0 & -\frac{\kappa_{PI}\theta_P\theta_I}{c_{P,I}}\theta_P \\ 0 & \frac{\text{diag}(\kappa_{CI}\delta V \ \theta_C\theta_I)}{c_{C,C}\delta V \delta V} + \frac{K_{\text{heat}}}{c_{C,C}\delta V} & -\frac{\kappa_{CI}\delta V \ \theta_I\theta_C}{c_{C,I}\delta V} \\ -\frac{\kappa_{PI}\theta_I\theta_P}{c_{I,P}} & -\frac{(\kappa_{CI}\delta V \ \theta_I\theta_C)^T}{c_{I,C}\delta V} & \frac{\kappa_{PI}\theta_P\theta_I + \theta_I \int \kappa_{CI}\theta_C dx}{c_{I,I}} \end{bmatrix} \begin{matrix} \theta_P \\ \theta_C \\ \theta_I \end{matrix}. \quad \text{(columns: } \theta_P, \ \theta_C, \ \theta_I\text{)} \tag{S1.3}$$

For brevity in our notation for the operators, we show only a subset of the rows and columns of the operators. The other entries not shown are taken to be zero. The input and output degrees of freedom of the operator are labeled using the last row (for input entries) and last column (for output entries). For example in $\bar{K}^{(1)}$, we show in the first row the entries associated with the $\mathbf{X}$ degrees of freedom and in the last row the entries associated with θ_P. We use a similar convention for the columns. The K_{heat} gives the heat exchange within the membrane, given by the operator $K_{\text{heat}} = -\nabla \cdot \left(\kappa_C \theta_C^2(x) \nabla\right)$. In practice, this is approximated in our finite volume discretization approach by $\tilde{K}_{\text{heat}}$ with

$$[\tilde{K}_{\text{heat}}]_{(i_0,j_0),(i_0,j_0)} = c\theta_{i_0,j_0} \left(\theta_{i_0+1,j_0} + \theta_{i_0-1,j_0} + \theta_{i_0,j_0+1} + \theta_{i_0,j_0-1}\right) \tag{S1.4}$$

$$[\tilde{K}_{\text{heat}}]_{(i_0\pm1,j_0),(i_0,j_0)} = -c\theta_{i_0,j_0}\theta_{i\pm1,j_0}, \tag{S1.5}$$

$$[\tilde{K}_{\text{heat}}]_{(i,j_0\pm1),(i_0,j_0)} = -c\theta_{i_0,j_0}\theta_{i_0,j\pm1}, \tag{S1.6}$$

where $c = \kappa_C/\Delta x^2$ and $\theta_{i,j} = \theta_C(x_{i,j})$. When this operator is applied to the gradient of the entropy $[\mathcal{D}\mathcal{S}]_{\theta_C(x)} = (c_C/\theta_C(x))\delta V$ this yields an approximation with the same action as the Laplacian to θ_C which is associated with the Fourier law of heat exchange. This provides for the model in equation 1– 4 the key terms needed in equation 7 to obtain the stochastic driving terms $\mathbf{g}^{(j)}$ for the fluctuations of the membrane-protein system.
