# Supplementary material for: Protein drift-diffusion in membranes with non-equilibrium fluctuations arising from gradients in concentration or temperature
Source: PLoS Comput Biol. 2025 Nov 21;21(11):e1013678. doi: 10.1371/journal.pcbi.1013678 (PMC12654922; doi:10.1371/journal.pcbi.1013678)
Supplement: S2 Appendix — (PDF) [file pcbi.1013678.s002.pdf]

S2. Stochastic Field Generation Methods and Factors $R^{(j)}$

We briefly give factorizations $R^{(j)}$ we have derived for generating the stochastic fields using equation 21. These can be verified to satisfy $R^{(j)}R^{(j),T} = K^{(j)}$ for $K^{(j)}$ given in the supplemental information in S1 Appendix. For the particle irreversible dynamics, we have

$$R^{(1)} = \begin{bmatrix} \sqrt{\theta_P} R_M(Y) \\ -\frac{\sqrt{\theta_P}\nabla_X \mathcal{E}^T R_M(Y)}{c_P} \end{bmatrix}, \tag{S2.1}$$

where $R_M R_M^T = \mathbf{M}_{XX}(\mathbf{Y})$. We generate the stochastic driving fields using

$$\mathbf{h}^{(1)} = R_1 \boldsymbol{\xi}_1. \tag{S2.2}$$

For the concentration field we have the factor

$$R^{(2)} = \begin{bmatrix} -\text{div}\left(\sqrt{\frac{q(x)\bar{\kappa}}{c_0}}\Box\right) & 0 \\ -\frac{c_0\nabla\Phi:\sqrt{\frac{q(x)\bar{\kappa}}{c_0}}\Box}{C_C} & -\text{div}\left(\sqrt{\frac{\bar{\kappa}_0\theta_C^2(x)}{C_C}}\Box\right) \end{bmatrix}. \tag{S2.3}$$

$$\mathbf{h}^{(2)} = R_1 \boldsymbol{\xi}_1. \tag{S2.4}$$

For the thermal exchanges of the interface coupling, we break the terms down into two parts $K^{(3)} = K_1^{(3)} + \int K_2^{(3)}(x)dx$. We use the factors

$$\begin{aligned} K_1^{(3)} &= \begin{bmatrix} \frac{\kappa_{PI}\theta_I\theta_P}{c_{P,P}} & -\frac{\kappa_{PI}\theta_P\theta_I}{c_{P,I}} \\ -\frac{\kappa_{PI}\theta_I\theta_P}{c_{I,P}} & \frac{\kappa_{PI}\theta_P\theta_I}{c_{I,I}} \end{bmatrix}_{\mathbf{e}_P,\mathbf{e}_I} \\ &= \kappa_{PI}\theta_P\theta_I\left[\frac{1}{C_{P,P}}\mathbf{e}_P\mathbf{e}_P^T - \frac{1}{C_{P,I}}\mathbf{e}_P\mathbf{e}_I^T - \frac{1}{C_{I,P}}\mathbf{e}_I\mathbf{e}_P^T + \frac{1}{C_{I,I}}\mathbf{e}_I\mathbf{e}_I^T\right] \\ &= R_1 R_1^T, \end{aligned} \tag{S2.5}$$

This just involves the parts of the operator with indices corresponding to θ_P, θ_I. We also use denote $C_{i,j} = C_i C_j$ to keep the notation consistent between cases. This has the factor

$$R_1 = \sqrt{\kappa_{PI}\theta_I\theta_P}\begin{bmatrix} \frac{1}{c_P}\mathbf{e}_P \\ -\frac{1}{c_I}\mathbf{e}_I \end{bmatrix}. \tag{S2.6}$$

The second part has similar factorization for each spatial location since $\kappa_{CI} = \kappa_{CI}(x)$ with

(S2.7)

$$
\begin{aligned}
K_2^{(3)} &= \begin{bmatrix} \frac{\mathrm{diag}\left(\kappa_{CI}\theta_I\theta_{C(x)}\ \delta V\right)}{c_{C,C}\delta V\delta V} & -\frac{\kappa_{CI}\theta_{C(x)}\theta_I\ \delta V}{c_{C,I}\delta V} \\ -\frac{\kappa_{CI}\theta_I\theta_{C(x)}\ \delta V}{c_{I,C}\delta V} & \frac{\int \kappa_{CI}\theta_{C(x)}\theta_I dx}{c_{I,I}} \end{bmatrix}_{\mathbf{e}_{\theta_{C(x)}},\mathbf{e}_{\theta_I}} \\
&= \int \kappa_{CI}(x)\theta_{C(x)}\theta_I \left[\frac{1}{c_{C,C}\delta V\delta V}\mathbf{e}_{\theta_{C(x)}}\mathbf{e}_{\theta_{C(x)}}^T - \frac{1}{c_{C,I}\delta V}\mathbf{e}_{\theta_{C(x)}}\mathbf{e}_{\theta_I}^T \right. \\
&+ \left. -\frac{1}{c_{I,C}\delta V}\mathbf{e}_{\theta_I}\mathbf{e}_{\theta_{C(x)}}^T + \frac{1}{c_{I,I}}\mathbf{e}_{\theta_I}\mathbf{e}_{\theta_I}^T\right] dx \\
&= \int \kappa_{CI}\theta_{C(x)}\theta_I \begin{bmatrix} \frac{1}{c_C\delta V}\mathbf{e}_{\theta_{C(x)}} \\ -\frac{1}{c_I}\mathbf{e}_{\theta_I} \end{bmatrix} \begin{bmatrix} \frac{1}{c_C\delta V}\mathbf{e}_{\theta_{C(x)}} \\ -\frac{1}{c_I}\mathbf{e}_{\theta_I} \end{bmatrix}^T dx \\
&= \int R_2(x)R_2^T(x)dx,
\end{aligned}
$$

where

$$
R_2(x) = \sqrt{\kappa_{CI}(x)\theta_I\theta_C(x)\delta V} \begin{bmatrix} \frac{1}{c_C\delta V}\mathbf{e}_{\theta_{C(x)}} \\ -\frac{1}{c_I}\mathbf{e}_{\theta_I} \end{bmatrix}. \quad \text{(S2.8)}
$$

We generate the stochastic driving fields for the fluctuations using

(S2.9)

$$
\mathbf{h}^{(3)} = \mathbf{h}_1 + \int \mathbf{h}_2(x)dx, \ \ \mathbf{h}_1 = R_1\boldsymbol{\xi}_1, \ \ \mathbf{h}_2(x) = R_2(x)\boldsymbol{\xi}_2(x).
$$

Since the $\boldsymbol{\xi}_{ij}(x_1), \boldsymbol{\xi}_{ij}(x_2)$ have zero correlation when $x_1 \neq x_2$, we have

(S2.10)

$$
\begin{aligned}
\langle \mathbf{h}_2\mathbf{h}_2^T\rangle &= \langle \mathbf{h}_1\mathbf{h}_1^T\rangle + \int \langle \mathbf{h}_2\mathbf{h}_2^T\rangle dx \\
&= R_1R_1^T + \int R_2(x)R_2(x)^T dx = K_1^{(3)} + \int K_2^{(3)}(x)dx = K^{(3)}.
\end{aligned}
$$

The expressions we have derived allow for avoiding the need to perform numerical Cholesky factorizations each time-step. These expressions allow for directly evaluating operators $R^{(j)}$ to generate the stochastic driving fields needed for sampling the system fluctuations.
