# Supplementary material for: Protein drift-diffusion in membranes with non-equilibrium fluctuations arising from gradients in concentration or temperature
Source: PLoS Comput Biol. 2025 Nov 21;21(11):e1013678. doi: 10.1371/journal.pcbi.1013678 (PMC12654922; doi:10.1371/journal.pcbi.1013678)
Supplement: S3 Appendix — Fig A in S3 Appendix. Transfer Operator Convergence. We show how the transfer operator for the temperature field θC(x) converges as the spatial discretization Δx is refined. We test the accuracy of u~(𝐱,t) from the numerical methods at different time steps t using the predicted solution u(𝐱,t). We consider the maximum error over the grid. We find the numerical methods exhibit second-order convergence O(Δx2) in agreement with theory. The spatial discretization Δx becomes smaller from left to right. The numerical tests were performed with default parameters in Table A in S3 Appendix. Fig B in S3 Appendix. Covariance of Increments of the Stochastic Time-Step Integrator. We show how the covariance of trajectories generated by the stochastic numerical methods compares with the target dynamics. Results are shown using a log scale. The numerical tests were performed with n = 104 samples of the integration step with the parameters in Table A in S3 Appendix. We found a maximum absolute error of ϵ=6.8×10^−9^. Table A in S3 Appendix. Parameters for the Stochastic Numerical Methods. We give the default values used in tests. (PDF) [file pcbi.1013678.s003.pdf]

S3. Validation of the Stochastic Numerical Methods

We perform a few tests to validate the stochastic numerical methods. This includes testing the transfer operators which are approximated using our spatial finite volume discretization approach in equations 19 - 20 and related methods discussed in the supplemental information in S1 Appendix. We perform convergence studies for how our discretizations approximate the continuum operators. For our stochastic time-step integration methods in equation 18, we perform tests of the generated dynamics. This includes testing the spatial-temporal covariance structure for the stochastic trajectories. The covariance structure provides an especially useful test since it depends on several different parts of the stochastic numerical methods performing correctly. The covariance and spatial approximation tests provide checks on both the theoretical properties of the numerical methods and the practical implementations.

**parameter**		**value**	**parameter**		**value**
κ_{PI}	heat conduction: particle	1.3×10^2	C_P	specific heat: particle	1.2
κ_{CI}	heat conduction: interface	1.0×10^2	C_C	specific heat: concentration	1.4
κ_{CC}	heat conduction: membrane	1.2×10^{-2}	C_I	specific heat: interface	1.3×10^2
κ_0	heat conduction: fluid	8.2×10^6	θ_0	baseline membrane temperature	3.0
c_0	total concentration	1.1	k_B	Boltzmann's constant	1.0×10^{-5}
n_x	number grid points in x	5.0	Δx	mesh spacing	1.0×10^{-1}
n_y	number grid points in y	5.0	Δt	time step	1.0×10^{-3}

S3 Table A. Parameters for the Stochastic Numerical Methods. We give the default values used in tests.

We validate the spatial discretizations of the continuum operators for transport within the membrane. We investigate the accuracy as the spatial discretization Δx for the mesh is refined. This impacts lateral transport of energy, such as in the temperature field θ_C given in equations 3. As a test function, we use the known analytic solution to the heat equation

$$u(x_1, x_2, t) = C_1 \exp\left(-\alpha_0 t\right) \sin(2\pi k_1 x_1 / L_1) \sin(2\pi k_2 x_2 / L_2) + C_2 \qquad \text{(S3.1)}$$
$$\alpha_0 = \frac{4\pi^2 \kappa_C}{L^2 c_C}\left(k_1^2 + k_2^2\right),$$

where $k_1 = k_2 = 2$, $C_1 = 3$, $C_2 = 6$. The default parameter values used in tests are given in S3 Table A. We take $\kappa_{CI} = 0$ to isolate the temperature field in this test, $L_1 = L_2 = L = 2.0$, and we vary n_x with $n_y = n_x$ to refine Δx. With the initial condition at $t = 0$, we make a comparison between the numerical solution obtained from the simulations $\tilde{u}$ with the predicted solution u. We consider the maximum error over the grid $\epsilon = \max_{ij} |\tilde{u}(\mathbf{x}_{ij}, t) - u(\mathbf{x}_{ij}, t)|$ where $\mathbf{x}_{ij} = \left((i + \frac{1}{2})\Delta x - L/2,\ \ (j + \frac{1}{2})\Delta x - L/2\right)$, $0 \leq i \leq n_x - 1$, and $0 \leq j \leq n_y$. We show results of our convergence tests in S3 Fig A.

We find the numerical methods exhibit second-order convergence $O(\Delta x^2)$. This validates the convergence of the numerical methods. This also validates their accuracy and the scaling of their error in agreement with theory. The results show the numerical methods provide accurate results both in discretizing the transport operators spatially and in their propagation over time.

We further test the stochastic numerical methods by performing simulations of the full system dynamics $Y(t)$ for equations 1- 4. We use Monte-Carlo sampling for the increments for a specified initial state $Y(0)$ to obtain $\Delta Y = Y(\Delta t) - Y(0)$. As discussed near equation 18, our stochastic time-step integration is a multi-stage

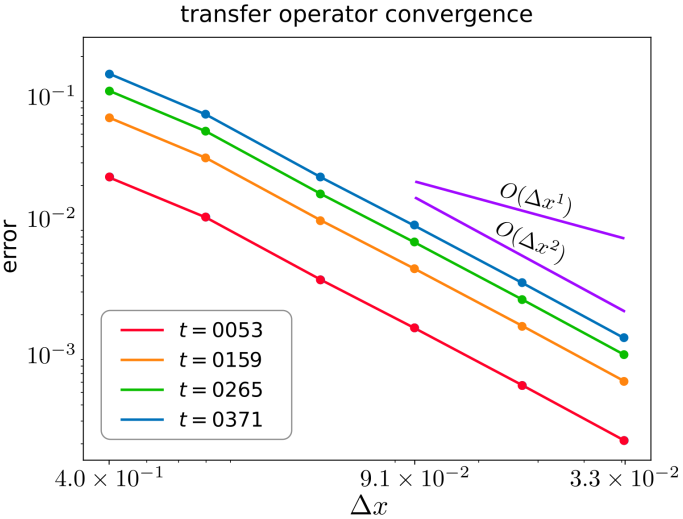

S3 Fig A. Transfer Operator Convergence. We show how the transfer operator for the temperature field $\theta_C(x)$ converges as the spatial discretization Δx is refined. We test the accuracy of $\tilde{u}(\mathbf{x}, t)$ from the numerical methods at different time steps t using the predicted solution $u(\mathbf{x}, t)$ in equation S3.1. We consider the maximum error over the grid. We find the numerical methods exhibit second-order convergence $O(\Delta x^2)$ in agreement with theory. The spatial discretization Δx becomes smaller from left to right. The numerical tests were performed with default parameters in Table A in S3 Appendix.

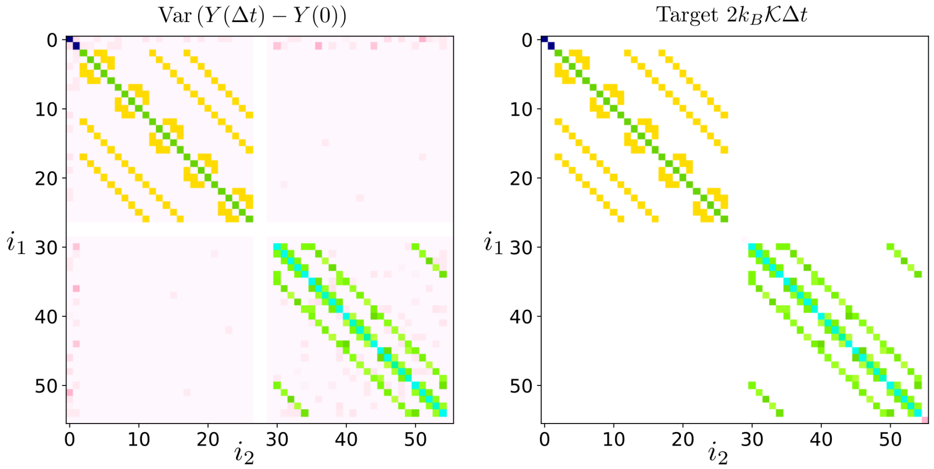

S3 Fig B. Covariance of Increments of the Stochastic Time-Step Integrator. We show how the covariance of trajectories generated by the stochastic numerical methods compares with the target dynamics. Results are shown using a log scale. The numerical tests were performed with $n = 10^4$ samples of the integration step with the parameters in Table A in S3 Appendix. We found a maximum absolute error of $\epsilon = 6.8 \times 10^{-9}$.

procedure designed to capture the drift-diffusion dynamics given in equations 6. From n samples of the stochastic trajectory generated by the numerical methods, we estimate the mean drift contributions as $\langle \Delta Y \rangle = \frac{1}{n}\sum_{i=1}^{n} \Delta Y^{(i)}$, where $\Delta Y^{(i)}$ is the ith sample.

To test the diffusive fluctuation contributions to the dynamics, we estimate the covariance contributions using

$$\mathrm{var}\left(\Delta Y\right) = \left\langle \Delta Y \Delta Y^T \right\rangle - \left(\left\langle \Delta Y \right\rangle\right)^2. \tag{S3.2}$$

We estimate the second moment using $\langle \Delta Y \Delta Y^T \rangle = \frac{1}{n}\sum_{i=1}^{n} \Delta Y^{(i)} \Delta Y^{(i),T}$. The spatial-temporal structure of the covariance depends on several different parts of the underlying stochastic numerical methods in order to perform correctly. The results also depend on our stochastic field generation methods based on our factorizations in supplemental information S2 Appendix. This requires that the multi-stage stochastic time integration correctly combine the fluctuating terms while preserving the temporal contributions. The numerical tests were performed with $n = 10^4$ samples of the integration step with the parameters in S3 Table A. For the initial state we use $\mathbf{X} = [5.0/3.0, 1.0]$, $q(x) = 1.0$, $\theta_I = 1.2$, $\theta_P = 3.0$. For the temperature field $\theta_C(x)$, we use the function in equation S3.1. We show the results in S3 Fig B.

The empirical studies show that the stochastic numerical methods yield the correct spatial-temporal covariance structure. It is found that the maximum absolute error for the tests is $\epsilon = 6.8 \times 10^{-9}$. These results show the analytic factorizations and related implementations are working correctly to provide accurate approximations. The results also further show that the multi-stage stochastic integration methods properly handle the stochastic terms. This samples multiple sources of fluctuations and captures their diffusive contributions to the system dynamics. In summary, the results show the numerical methods and implementations are able to provide an accurate approximation of the continuum spatial operators and the stochastic dynamics of the system.
